# Influence of baseline arterial stiffness on effects of intensive compared with standard blood pressure control: a post hoc analysis of the STEP trial

**DOI:** 10.1186/s12916-022-02556-1

**Published:** 2022-10-20

**Authors:** Qirui Song, Qianhui Ling, Jingjing Bai, Hongwei Zhang, Peili Bu, Fang Chen, Shouling Wu, Weili Zhang, Mulei Chen, Jun Cai

**Affiliations:** 1 Hypertension Center, Fuwai Hospital, State Key Laboratory of Cardiovascular Disease of China, National Center for Cardiovascular Diseases of China, Chinese Academy of Medical Sciences and Peking Union Medical College, Beilishi Rd. 167, Xicheng District, Beijing, 100037 China; 2 The Hospital of Shunyi District Beijing, Beijing, 101300 China; 3 Qilu Hospital of Shandong University, Jinan, 250012 Shandong China; 4 The First People’s Hospital of Yinchuan, Yinchuan, 750000 Ningxia China; 5 Kailuan General Hospital, Tangshan, 063000 Hebei China; 6 Heart Center and Beijing Key Laboratory of Hypertension, Department of Cardiology, Beijing Chaoyang Hospital, Capital Medical University, Gongtinanlu Rd. 8, Chaoyang District, Beijing, 100020 China

**Keywords:** Vascular stiffness, Hypertension, Intensive BP lowering

## Abstract

**Background:**

The benefits and risks of intensive versus standard systolic blood pressure (SBP) treatment in older patients with arterial stiffness (AS) remains unclear. This study aims to investigate the interaction between the baseline AS and SBP treatments on cardiovascular outcomes.

**Methods:**

In this post hoc analysis of the Strategy of Blood Pressure Intervention in the Elderly Hypertensive Patients (STEP) trial, we involved 6865 participants with complete data regarding baseline brachial-ankle pulse wave velocity (baPWV). Patients were categorized by baseline AS status (AS, baPWV ≥ 1800 cm/s; non-AS, baPWV < 1800 cm/s). The primary outcome was a composite of cardiovascular events. The secondary outcomes were stroke, acute coronary syndrome (ACS), major cardiovascular events (MACE), and all-cause death. Cox regression was used to calculate hazard ratios for the outcomes.

**Results:**

During a mean follow-up of 2.69 years, a total of 248 primary outcome events and 81 all-cause deaths occurred. The hazard ratios for the primary outcome were 0.76 (95% confidence interval (CI), 0.54–1.09) and 0.63 (95% CI, 0.43–0.92) in the AS and non-AS groups, respectively (P for interaction = 0.43), and that for stroke was 0.58 (95% CI, 0.33–1.02) and 0.48 (95% CI, 0.23–0.99) in the AS and non-AS groups, respectively (*P* for interaction = 0.68). Effects of intensive SBP treatment on safety outcomes and all-cause death were also similar in the two groups (*P* for interaction > 0.05 for all).

**Conclusions:**

In the STEP trial, the beneficial effects of intensive SBP treatment were similar among those in the AS group and the non-AS group at baseline.

**Trial registration:**

STEP ClinicalTrials.gov number, NCT03015311. Registered 2 January 2017.

**Supplementary Information:**

The online version contains supplementary material available at 10.1186/s12916-022-02556-1.

## Background

Hypertension is the leading modifiable risk factor for cardiovascular morbidity and mortality [1]. Cardiovascular diseases, mainly ischemic heart disease and stroke, are the leading cause of death globally [2]. Emerging evidence has proven the benefit of intensive systolic blood pressure (SBP) control among patients with hypertension [3, 4]. The Strategy of Blood Pressure Intervention in the Elderly Hypertensive Patients (STEP) trial recently showed that an intensive SBP target of 110 to <130 mmHg reduced the risk of cardiovascular diseases and stroke by 26% and 33%, respectively, compared with an SBP target of 130 to <150 mmHg [5].

Arterial stiffness (AS) and hypertension frequently coexist, particularly in older patients. AS, a characteristic feature of aging arteries [6], is an independent age-related risk factor for stroke, coronary heart disease, cardiovascular death, and all-cause death in patients with hypertension [7–9]. Patients with AS and hypertension have a two-fold higher risk of cardiovascular events than patients with non-AS and normotension [10]. Patients with AS tend to have impaired arterial compliance and loss of ability to dampen the pulsatility of ventricular ejection, resulting in increased SBP and decreased diastolic blood pressure (DBP) [11, 12]. Because previous studies suggested a J-curve relationship between DBP and cardiovascular events [13–17], it is of concern whether patients with AS are susceptible to insufficient organ perfusion caused by low DBP when achieving the intensive SBP target. Whether the effect of intensive versus standard SBP treatment is different in patients with and without AS is unclear.

We initiated this post hoc analysis of the STEP trial to investigate the interaction between the baseline AS status and SBP treatments on cardiovascular outcomes, risk of low on-treatment DBP, and safety outcomes. AS was assessed using the brachial-ankle pulse wave velocity (baPWV), a reproducible, noninvasive, and convenient clinical test that is well-validated in the Asian population and shows a predictive value similar to that of the carotid-femoral aortic pulse wave velocity (cfPWV) [18, 19].

## Methods

### Study design and population

This study was a post hoc analysis of the STEP trial (STEP ClinicalTrials.gov number, NCT03015311). Details regarding the design, rationale, and primary outcomes of the STEP trial have been published previously [3, 5]. Briefly, the STEP trial was an open-label, multicenter, randomized controlled trial that compared the effects of intensive (SBP target of 110 to <130mmHg) and standard (SBP target of 130 to <150 mmHg) SBP treatment on cardiovascular outcomes in 8511 patients with hypertension at 42 clinical centers in China. The study was approved by the Ethics Committee of Fuwai Hospital and all collaborating centers. All participants provided written informed consent.

**Fig. 1:**
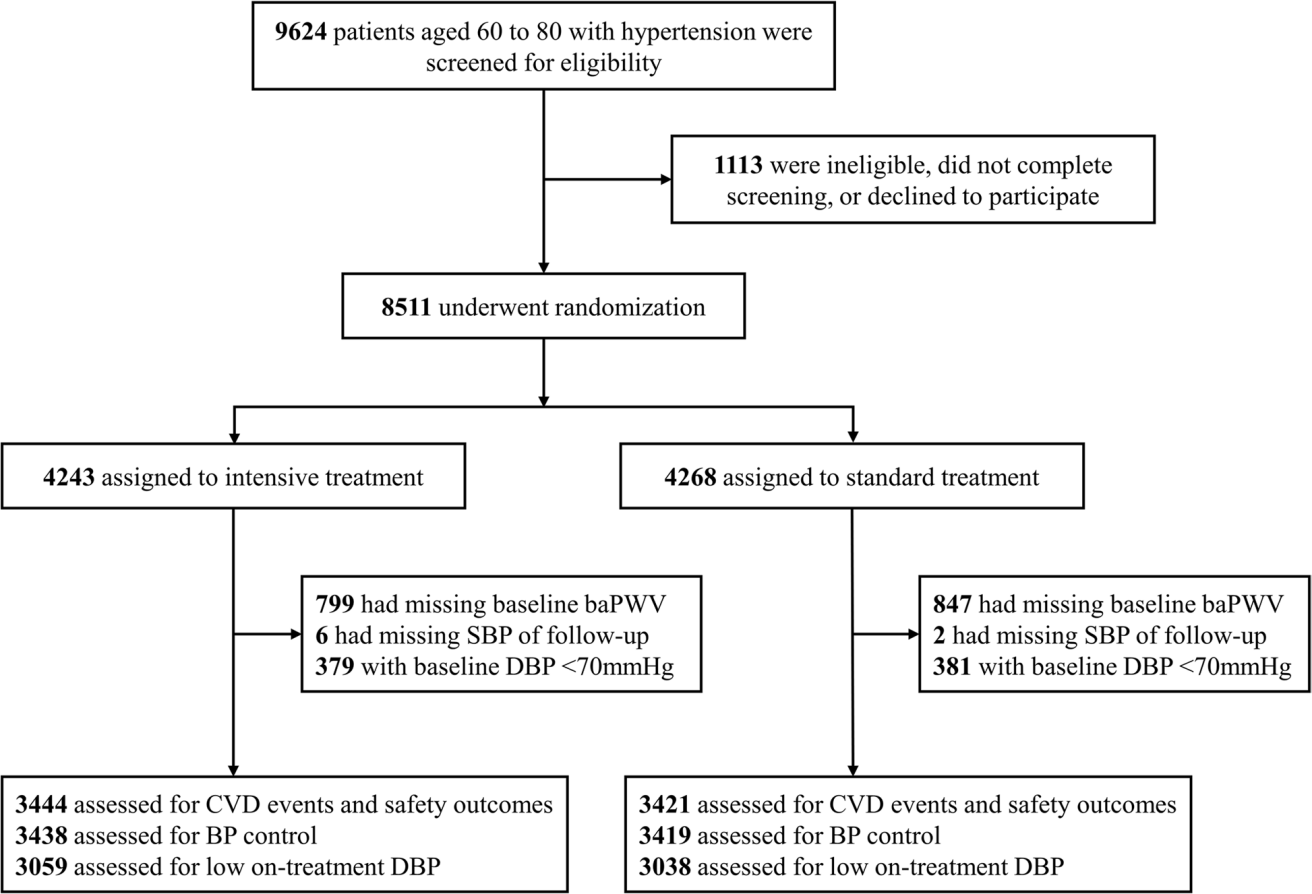
Flow chart of patient selection. Abbreviations: baPWV, brachial-ankle pulse wave velocity; SBP, systolic blood pressure; BP, blood pressure; CVD, cardiovascular disease. Median follow-up, 2.69 years

The inclusion and exclusion criteria were identical to those in the STEP trial. Eligible participants included those aged 60 to 80 years with essential hypertension, defined as SBP of 140 to 190 mmHg, or currently taking antihypertensive medication. Patients with a history of ischemic or hemorrhagic stroke were excluded. Moreover, in this present analysis, 1646 patients who had missing data regarding baseline baPWV and 8 patients with missing BP data during follow-up were excluded. Furthermore, 760 patients with baseline DBP < 70mmHg were excluded for the analysis of the incidence of low on-treatment DBP. Finally, 6865 participants were included in the analysis of cardiovascular outcomes and safety outcomes, 6857 were included in the analysis of BP control, and 6097 were included in the analysis of low on-treatment DBP (Fig. 1).

### Randomization and Intervention

Eligible participants were randomly assigned to either the intensive treatment group (SBP target of 110 to <130 mmHg) or the standard treatment group (SBP target of 130 to <150 mmHg) by a central computerized randomization program. After randomization, all participants were scheduled for follow-up once monthly for the first 3 months and every 3 months thereafter until the final visit or death. A detailed antihypertensive treatment algorithm to reach these BP targets has been previously published [5].

### Ascertain of AS

To assess AS at baseline, baPWV was measured with an Omron BP-203RPEIII automatic waveform analyzer (Omron Healthcare, Kyoto, Japan). After the participants had rested for at least 5 minutes in the supine position at an ambient temperature of 22°C to 25°C, trained trial staff placed electrodes on the participants’ wrists, placed a microphone on the left edge of the sternum, and placed pneumatic cuffs on both arms and ankles. The lower edge of the brachial cuff was positioned 2 to 3 cm above the transverse striation of the cubital fossa, and the lower edge of the ankle cuff was positioned 1 to 2 cm above the superior aspect of the medial malleolus. Sensors were used to determine the volume pulse form and BP, and a semiconductor pressure sensor was used to record the pulse volume waveforms. For each participant, measurements were conducted twice, and the latter measurement was recorded. The maximum values of the left- and right-sided baPWV were used for analysis.

The patients were divided into AS and non-AS groups according to their baseline baPWV. The AS group comprised patients with a baseline baPWV of ≥1800 cm/s, and the non-AS group comprised patients with a baseline baPWV of <1800 cm/s [20].

### Covariates

The sociodemographic characteristics of each participant were collected by trained STEP physicians at baseline, including age, sex, body mass index, physical activity (never, 1 to 2 times/week, or ≥3 times/week), and smoking and drinking frequency (never, ever, or current). The 10-year risk of cardiovascular disease was estimated using the Framingham risk score [21]. Current smokers were defined as those who smoked at least one cigarette per day for more than 6 months. Current alcohol drinkers were defined as those who drank at least once per month for more than 6 months. Physical activity was evaluated according to the type and frequency of physical activity at work and during leisure time and was categorized as never, 1 to 2 times/week, or ≥3 times/week. Body mass index was calculated by dividing the weight (kg) by the square of the height (m).

Clinical information including office BP was collected at baseline and every 3 months during the follow-up period. At each visit, office BP was measured using the same validated automatic BP monitor device (Omron HBP-1100U; Omron Healthcare). The participants were required to rest for at least 5 minutes before BP measurement. A trained physician or nurse performed BP measurements three times at an interval of 1 to 2 minutes, and the average value was recorded. Laboratory examinations, including measurement of the creatinine, fasting blood glucose, triglyceride, total cholesterol, high-density lipoprotein cholesterol, and low-density lipoprotein cholesterol concentrations, were performed at baseline and yearly thereafter.

### Definition of outcomes

The primary outcome of the present study was an incident cardiovascular event, including a composite of the first occurrence of stroke (ischemic or hemorrhagic), acute coronary syndrome (myocardial infarction and hospitalization for unstable angina), acute decompensated heart failure, coronary revascularization (percutaneous coronary intervention or coronary artery bypass grafting), atrial fibrillation, or death of cardiovascular causes. This primary outcome was identical to that in the STEP trial [5]. The secondary outcomes were stroke, major cardiovascular events (MACE), acute coronary syndrome (ACS), and death of any cause. MACE was defined as a composite of the first occurrence of acute coronary syndrome, acute decompensated heart failure, coronary revascularization, and death of cardiovascular causes. The BP outcome in this study was the risk of low on-treatment DBP, which was defined as a mean achieved DBP of <70 mmHg during follow-up because this threshold has long been regarded as harmful with respect to cardiovascular outcomes [16, 22].

The safety outcomes were hypotension, dizziness, syncope, fracture, and the renal outcome. The renal outcome was a composite of a ≥50% decrease in the estimated glomerular filtration rate (eGFR) in patients with chronic kidney disease (CKD) at baseline, a ≥30% decrease in the eGFR to <60 mL/min/1.73 m^2^ in patients without CKD at baseline, or a serum creatinine increase of >1.5 mg/dL in men or >1.3 mg/dL in women. The eGFR was calculated using the Modification of Diet in Renal Disease equation [23]. CKD was defined as an eGFR of <60 mL/min/1.73 m^2^.

### Statistical methods

Among 6865 patients in this study, 3047 patients were divided into the AS group, and 3818 patients were divided into the non-AS group. The absolute risk of the primary outcome in the non-AS group was 3.06%. Using this number of patients and a two-tailed alpha of 0.05, this study had 80% power to detect a 26% relative change in the hazard ratio of AS group compared with the non-AS group. Moreover, based on 3444 patients in the intensive treatment group and 3421 in the standard treatment group enrolled in this study and an absolute risk of 4.24% in the standard group for the primary outcome, this post hoc analysis provided 80% power to detect a 20% relative risk reduction in the primary events between intensive and standard SBP treatment. The statistical power was calculated using PASS (version 15).

Continuous variables are presented as mean ± standard deviation, and categorical variables are presented as *n* (%). Baseline characteristics were compared across baseline baPWV groups (AS and non-AS) and across SBP treatment groups stratified by baseline baPWV groups using one-way analysis of variance for continuous variables and the chi-square (*χ*^2^) test for categorical variables. The mean follow-up BP status was calculated for each patient by averaging his or her BP measurements from month 3 to the last visit.

We analyzed the association between the baseline baPWV groups (AS and non-AS) and the primary and secondary outcomes using Cox proportional hazards regression. We then used the Fine–Gray subdistribution hazard model to calculate the hazard ratios (HRs) with 95% confidence intervals (CIs) for the primary outcome and secondary outcomes except for death from any cause associated with intensive SBP treatment versus standard SBP treatment (reference) among participants in the AS or non-AS group. For death from any cause, the Cox regression model was used. To assess the interaction effect of the SBP treatment group among patients with AS and non-AS, the product term (SBP treatment group × AS or non-AS) was included in the Cox proportional hazards regression models with a likelihood ratio test. The proportional hazards assumption was tested by Schoenfeld residuals for AS status, SBP intervention, and all covariates, and no violations were observed. All multivariable models were adjusted for SBP intervention, clinical centers, age, and sex, baseline mean arterial pressure (MAP) level, baseline glucose level, baseline LDL cholesterol level, baseline antihypertensive agent type, physical activity frequency, smoking status, and drinking status. In addition, cumulative incidence curves were performed using a Kaplan–Meier survival curve to compare the incidence of the primary outcome and stroke between the intensive and standard treatment arms within the AS and non-AS groups.

We analyzed the effect of the baseline AS status on the incidence of low on-treatment DBP (<70 mmHg) and safety outcomes using a logistic regression model adjusted for SBP intervention, clinical centers, age, and sex, baseline mean arterial pressure (MAP) level, baseline glucose level, baseline LDL cholesterol level, baseline antihypertensive agent type, physical activity frequency, smoking status, and drinking status. Similar analyses were performed to investigate the interactions between the SBP treatment group and baseline AS status for the incidence of low on-treatment DBP (<70 mmHg) and safety outcomes based on the logistic regression model. The odds ratio (OR) was calculated by the exponential of the coefficient.

All analyses were performed using R version 4.1.2. A two-sided *P* value of <0.05 was considered statistically significant. Missing values for the smoking status, drinking status, and baseline laboratory examinations were added via multiple imputation (mice 3.14.0).

## Results

### Baseline characteristics

Among the 6865 participants included in this study (mean age, 66.14 ± 4.75 years; 53.55% female), 3444 were assigned to the intensive treatment group and 3421 to the standard group (Fig. 1). The mean baseline baPWV in the AS and non-AS groups was 2079.25±236.28 and 1553.19±174.64 cm/s, respectively. The participants’ baseline characteristics were compared between the intensive and standard treatment groups in different baPWV groups (Table 1). Patients receiving the intensive treatment had higher ratios of angiotensin receptor blocker and calcium channel blocker use at baseline. No significant differences were observed between the different interventions within both AS and non-AS groups in other features. The baseline characteristics were also compared between AS and non-AS groups (Table 1). Patients in the AS group had a higher age, baseline SBP, PP, MAP, fasting blood glucose concentration, and triglyceride concentration and a lower eGFR than those in the non-AS group. The AS group contained more patients with an eGFR of <60 mL/min/1.73 m^2^, history of diabetes, and high 10-year risk of cardiovascular disease (estimated by Framingham risk score of ≥15%). The baseline DBP, activity frequency, plasma lipid concentration, and other laboratory examination findings were similar between the two groups. The baseline characteristics of participants included and excluded in this analysis were stated in Additional file 1: Table S1.

**Table 1:** Baseline characteristics between intensive and standard treatment groups within AS and non-AS groups

	Non-AS			AS			Non-AS	AS	***P*** value
	Intensive treatment	Standard treatment	***P*** value	Intensive treatment	Standard treatment	***P*** value			
**N (%)**	1980 (51.86)	1838 (48.14)		1464 (48.05)	1583 (51.95)		3818 (55.62)	3047 (44.38)	
**Age, years**	65.1±4.25	65.14±4.38	0.77	67.44±4.98	67.42±4.94	0.91	65.12±4.32	67.43±4.96	<0.001
**Female,** ***n*** **(%)**	1053 (53.18)	920 (50.05)	0.06	789 (53.89)	914 (57.74)	0.04	1973 (51.68)	1703 (55.89)	<0.001
**BMI, kg/m**^**2**^	25.76±3.19	25.85±3.2	0.36	25.34±3.14	25.41±3.15	0.53	25.8±3.19	25.38±3.14	<0.001
**HR, bpm**	72.8±10.17	72.18±9.99	0.06	74.79±10.71	74.93±10.65	0.71	72.5±10.09	74.86±10.68	<0.001
**BP, mmHg**									
**SBP**	143.78±16.07	143.43±16.03	0.50	150.4±17.38	149.92±16.69	0.44	143.61±16.05	150.15±17.03	<0.001
**DBP**	82.6±10.38	82.58±10.26	0.95	82.93±11.1	82.28±10.62	0.09	82.59±10.32	82.59±10.86	0.99
**PP**	61.18±13.64	60.85±13.96	0.46	67.46±15.01	67.64±14.51	0.74	61.02±13.79	67.56±14.75	<0.001
**MAP**	102.99±10.79	102.86±10.61	0.70	105.42±11.52	104.82±11.02	0.14	102.93±10.7	105.11±11.26	<0.001
**baPWV, cm/s**	1562.24±171.74	1544.79±176.92	0.002	2080.5±239.06	2078.08±233.75	0.78	1553.19±174.64	2079.25±236.28	<0.001
**Physical activity, times/week,** ***n*** **(%)**			0.22			0.86			0.06
**≥ 3**	1232 (62.22)	1193 (64.91)		963 (65.78)	1055 (66.65)		2425 (63.51)	2018 (66.23)	
**1–2**	429 (21.67)	374 (20.35)		285 (19.47)	297 (18.76)		803 (21.03)	582 (19.10)	
**0**	319 (16.11)	271 (14.74)		216 (14.75)	231 (14.59)		590 (15.45)	447 (14.67)	
**Smoking status,** ***n*** **(%)**			0.19			0.16			<0.001
**Current**	325 (16.44)	342 (18.66)		222 (15.22)	203 (12.86)		667 (17.51)	425 (13.99)	
**Former**	246 (12.44)	226 (12.33)		158 (10.83)	182 (11.53)		472 (12.39)	340 (11.19)	
**Never**	1406 (71.12)	1265 (69.01)		1079 (73.95)	1194 (75.62)		2671 (70.10)	2273 (74.82)	
**Drink status,** ***n*** **(%)**			0.02			0.52			<0.001
**Current**	531 (26.86)	541 (29.51)		362 (24.81)	365 (23.12)		1072 (28.14)	727 (23.93)	
**Former**	91 (4.60)	108 (5.89)		76 (5.21)	80 (5.07)		199 (5.22)	156 (51.35)	
**Never**	1355 (68.54)	1184 (64.59)		1021 (69.98)	1134 (71.82)		2539 (66.64)	2155 (70.93)	
**FBG, mmol/L**	6.02±1.44	5.99±1.52	0.57	6.26±1.62	6.35±1.72	0.11	6±1.48	6.31±1.67	<0.001
**TC, mmol/L**	4.84±1.04	4.91±1.12	0.07	4.9±1.12	4.93±1.09	0.52	4.88±1.08	4.91±1.1	0.18
**LDL-C, mmol/L**	2.7±0.86	2.72±0.87	0.55	2.68±0.88	2.71±0.89	0.49	2.71±0.87	2.69±0.89	0.55
**HDL-C, mmol/L**	1.26±0.3	1.26±0.31	0.69	1.26±0.31	1.26±0.31	0.58	1.26±0.3	1.26±0.31	0.97
**TG, mmol/L**	1.53±0.96	1.59±1.17	0.07	1.63±1.1	1.63±1.1	0.92	1.56±1.08	1.63±1.1	0.01
**UA, mmol/L**	346.85±87.7	346.23±89.01	0.83	349.87±88.98	346.17±89.88	0.26	346.53±88.37	347.94±89.45	0.52
**eGFR, ml/min**	109.2±22.57	110.34±23.07	0.13	108.42±25.12	108.32±24.58	0.92	109.79±22.83	108.37±24.84	0.01
**eGFR<60,** ***n*** **(%)**	23 (1.19)	24 (1.34)	0.80	32 (2.24)	32 (2.07)	0.85	47 (1.27)	64 (2.15)	0.006
**Medical history,** ***n*** **(%)**									
**Diabetes mellitus**	322 (16.26)	301 (16.38)	0.96	356 (24.32)	366 (23.12)	0.46	623 (16.32)	722 (23.70)	<0.001
**CVD History**	372 (18.79)	339 (18.44)	0.82	252 (17.21)	256 (16.17)	0.47	711 (18.62)	508 (16.67)	0.04
**Antihypertensive agents**									
**No. of antihyperten-sive agents**	1.39±0.63	1.52±0.68	<0.001	1.56±0.68	1.44±0.69	<0.001	1.46±0.66	1.5±0.69	0.02
**Not using antihyper-tensive agents,** ***n*** **(%)**	66 (3.33)	59 (3.21)	0.90	51 (3.48)	65 (4.11)	0.42	125 (3.27)	116 (3.81)	0.26
**Use of ARB,** ***n*** **(%)**	1273 (64.29)	1083 (58.92)	0.001	960 (65.57)	926 (58.50)	<0.001	2356 (61.71)	1886 (61.90)	0.89
**Use of CCB,** ***n*** **(%)**	1502 (75.86)	1313 (71.44)	0.002	1155 (78.89)	1198 (75.68)	0.04	2815 (73.73)	2353 (77.22)	<0.001
**Use of statins,** ***n*** **(%)**	439 (22.17)	392 (21.33)	0.55	247 (16.87)	269 (16.99)	0.97	831 (21.77)	516 (16.93)	<0.001
**Use of aspirin,** ***n*** **(%)**	196 (9.90)	182 (9.90)	1.00	109 (7.45)	134 (8.46)	0.33	378 (9.90)	243 (7.98)	0.007
**FRS ≥15%,** ***n*** **(%)**	1427 (74.40)	1328 (74.61)	0.92	1210 (85.03)	1268 (82.39)	0.06	2755 (74.50)	2478 (83.66)	<0.001

Abbreviations: *AS* arterial stiffness, *BMI* body mass index, *HR* heart rate, *SBP* systolic blood pressure, *DBP* diastolic blood pressure, *PP* pulse pressure, *MAP* mean arterial pressure, *baPWV* brachial-ankle pulse wave velocity, *FBG* fasting blood glucose, *TC* total cholesterol, *LDL-C* low-density lipoprotein cholesterol, *HDL-C* high-density lipoprotein cholesterol, *TG* triglyceride, *UA* uric acid, *eGFR* estimated glomerular filtration rate, *CVD* cardiovascular disease, *ARB* angiotensin receptor blocker, *CCB* calcium channel blocker, *FRS* Framingham risk score

### Interactions of baseline baPWV and SBP intervention for prespecified cardiovascular outcomes

In total, 248 primary outcome events and 81 all-cause deaths (including 24 cardiovascular deaths) occurred during a median follow-up of 2.69 ± 0.67 years. The incidences of the primary outcome and stroke, and MACE were significantly lower in the intensive treatment group than in the standard treatment group (Additional file 1: Table S2).

The incidence of stroke was significantly higher in the AS group than in the non-AS group (HR, 1.70; 95% CI, 1.09–2.65) after multivariable adjustment (Additional file 1: Table S3). The incidences of the primary outcome and all-cause death were higher in the AS group in the crude model (primary outcome: HR, 1.40; 95% CI, 1.09–1.79 and all-cause death: HR, 1.64; 95% CI, 1.06–2.55) (Additional file 1: Table S3). However, they became insignificant after adjusting for confounders (primary outcome: HR, 1.26; 95% CI, 0.97–1.63 and all-cause death: HR, 1.21; 95% CI, 0.70–1.92) (Additional file 1: Table S3). The effect of per SD change in baseline baPWV on the incidence of the primary outcome, stroke, and all-cause death were similar with that of baseline AS versus non-AS groups (Additional file 1: Table S4).

**Fig. 2:**
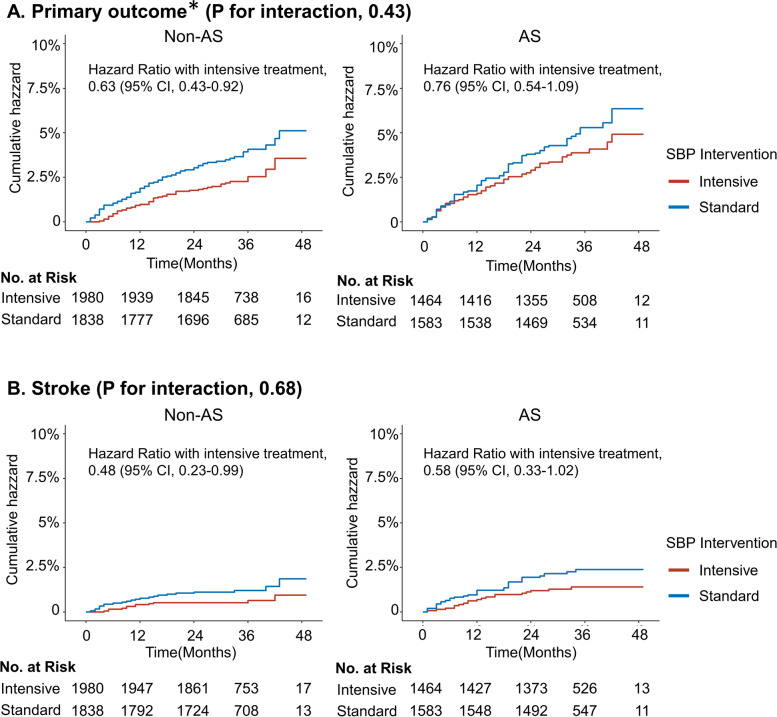
Cumulative incidence of **A** primary outcome and **B** stroke by treatment arm stratified by patients within the AS and non-AS groups at baseline. Abbreviations: AS, arterial stiffness; SBP, systolic blood pressure; CI, confidence interval. *Primary outcome contains the first occurrence of stroke (ischemic or hemorrhagic), acute coronary syndrome (myocardial infarction and hospitalization for unstable angina), acute decompensated heart failure, coronary revascularization (percutaneous coronary intervention or coronary artery bypass grafting), atrial fibrillation, or death of cardiovascular causes

The incidences of the primary outcome, stroke, MACE, ACS, and all-cause death between intensive and standard SBP treatment across the AS and non-AS groups are presented in Table 2. The HR of intensive versus standard SBP treatment for the primary outcome was 0.76 (95% CI, 0.54–1.09) and 0.63 (95% CI, 0.43–0.92) among patients in the AS and non-AS groups, respectively (*P* for interaction = 0.43) (Fig. 2, Table 2). Similarly, the HR for stroke was 0.58 (95% CI, 0.33–1.02) and 0.48 (95% CI, 0.23–0.99) among those in the AS and non-AS groups, respectively (*P* for interaction = 0.68) (Fig. 2, Table 2). The HR for MACE was 0.83 (95% CI, 0.53–1.29) and 0.62 (95% CI, 0.39–0.97) among those in the AS and non-AS groups, respectively (*P* for interaction = 0.34) (Table 2). There was no significant association of ACS or all-cause death with SBP interventions.

**Table 2:** Effects of SBP intervention on cardiovascular outcomes within AS and non-AS groups via multivariable Cox proportional hazard regression

	Non-AS				AS				***P*** for interaction^*^
	Intensive treatment	Standard treatment	Hazard ratio (95% CI)	***P*** value	Intensive treatment	Standard treatment	Hazard ratio (95% CI)	***P*** value	
***N***	1980	1838			1464	1583			
**Primary outcome**^**a**^**,** ***n*** **(%)**	48 (2.42)	69 (3.75)	0.63 (0.43–0.92)	0.02	55 (3.76)	76 (4.80)	0.76 (0.54–1.09)	0.13	0.43
**Stroke,** ***n*** **(%)**	12 (0.61)	23 (1.25)	0.48 (0.23–0.99)	0.05	19 (1.30)	35 (2.21)	0.58 (0.33–1.02)	0.06	0.68
**MACE**^**b**^**,** ***n*** **(%)**	36 (1.82)	51 (2.77)	0.62 (0.39–0.97)	0.04	37 (2.53)	46 (2.91)	0.83 (0.53–1.29)	0.40	0.34
**ACS,** ***n*** **(%)**	21 (1.06)	29 (1.58)	0.66 (0.39–1.19)	0.17	21 (1.43)	26 (1.64)	0.87 (0.48–1.58)	0.66	0.52
**Death from any causes,** ***n*** **(%)**	21 (1.06)	14 (0.76)	1.41 (0.71–2.78)	0.33	22 (1.50)	24 (1.52)	0.98 (0.55–1.76)	0.96	0.48

Multivariable model was adjusted for clinical centers, age, and sex, baseline MAP level, baseline glucose level, baseline LDL cholesterol level, baseline antihypertensive agent type, physical activity frequency, smoking status, and drinking status. Missing values of physical activity frequency, smoking status, drinking status (*n* = 17, 0.26%), baseline glucose level (*n*=214, 3.12%), baseline LDL cholesterol level (*n*=178, 2.59%) were added via multiple imputation

Abbreviations: *AS* arterial stiffness, *SBP* systolic blood pressure, *CI* confidence interval, *ACS* acute coronary syndrome, *MACE* major adverse cardiac events

^*^P for interaction was calculated by the multiplicative interaction between the baseline AS and non-AS groups and SBP intervention for the incidence of primary and secondary outcomes

^a^Primary outcome contains the first occurrence of stroke (ischemic or hemorrhagic), acute coronary syndrome (myocardial infarction and hospitalization for unstable angina), acute decompensated heart failure, coronary revascularization (percutaneous coronary intervention or coronary artery bypass grafting), atrial fibrillation, or death of cardiovascular causes

^b^MACE was a composite of first occurrence of acute coronary syndrome, acute decompensated heart failure, coronary revascularization, and death of cardiovascular causes

There was no suggestion of heterogeneity of the hazard ratios for intensive SBP treatment versus standard SBP treatment on the primary outcome and prespecified secondary outcomes within the AS and non-AS groups. All *P* values for interaction between the SBP treatment effect and baseline baPWV groups were >0.05 (Table 2).

### Interactions of baseline baPWV and SBP intervention for safety outcomes

The incidences of the safety outcomes did not differ significantly between the intensive and standard SBP intervention groups with the exception of hypotension (HR, 1.37; 95% CI, 1.05–1.79) (Additional file 1: Table S2).

The incidence of hypotension was lower and the incidence of fracture was higher in the AS group than in the non-AS group. No differences were found in dizziness, syncope, or renal outcomes between the AS and non-AS groups (Additional file 1: Table S5).

The incidence of safety outcomes (including hypotension, dizziness, fracture, syncope, and renal outcomes) is summarized in Additional file 1: Table S6. The *P* values for interaction between the SBP treatment group and baseline baPWV groups were >0.05 for all safety outcomes. Heterogeneity was not detected of the intensive SBP treatment on safety outcomes among patients in the AS and non-AS groups (Additional file 1: Table S6).

### Interactions of baseline baPWV and SBP intervention for low on-treatment DBP

There was an increased incidence of low on-treatment DBP in the intensive treatment group compared to the standard group after adjusting for confounders (OR, 3.34; 95% CI, 2.51–4.45; *P* < 0.001) (Additional file 1: Table S2). In the standard treatment group, the incidence of low on-treatment DBP was higher in the AS group compared with non-AS group in the crude model (OR, 1.95; 95% CI, 1.23–3.14; *P*=0.005) while insignificant after adjusting for confounders (OR, 1.56; 95% CI, 0.92–2.63; *P*=0.10). In the intensive treatment group, no significant differences were found in the incidence of low on-treatment DBP between the AS and non-AS groups before (OR, 1.02; 95% CI, 0.77–1.35; *P*=0.87) and after adjustment (OR, 0.96; 95% CI, 0.71–1.32; *P*=0.82) (Table 3).

**Table 3:** Effect of baseline baPWV and SBP intervention on the incidence of low on-treatment DBP (<70 mmHg)

Intervention	On-treatment DBP <70 mmHg	% On-treatment DBP <70 mmHg	Odds ratio (95% CI)			
			Univariable model	***P*** value	Multivariable model	***P*** value
**Intensive treatment**						
**Non-AS**	122/1760	6.93	Reference	0.87	Reference	0.82
**AS**	92/1299	7.08	1.02 (0.77–1.35)		0.96 (0.71–1.32)	
**Standard treatment**						
**Non-AS**	29/1631	1.78	Reference	0.005	Reference	0.10
**AS**	48/1407	3.41	1.95 (1.23–3.14)		1.56 (0.92–2.63)	
***P*** for interaction*			0.02		0.07	

Multivariable model was adjusted for clinical centers, age, and sex, baseline MAP level, baseline glucose level, baseline LDL cholesterol level, baseline antihypertensive agent type, physical activity frequency, smoking status, and drinking status. Missing values of physical activity frequency, smoking status, drinking status (*n* = 17, 0.26%), baseline glucose level (*n*=214, 3.12%), baseline LDL cholesterol level (*n*=178, 2.59%) were added via multiple imputation

Abbreviations: *baPWV* brachial-ankle pulse wave velocity, *SBP* systolic blood pressure, *DBP* diastolic blood pressure, *MAP* Mean arterial pressure, *CI* confidence interval, *AS* arterial stiffness, *LDL* low-density lipoprotein

^*^P for interaction was calculated by the multiplicative interaction between the baseline AS and non-AS groups and SBP intervention for the incidence of DBP < 70 mmHg

## Discussion

The results of the present study suggest that the beneficial effects of intensive SBP treatment were similar among those in AS group and non-AS group at baseline. Notably, the intensive SBP treatment consistently reduced the risk of stroke among patients with and without AS, although baseline AS was associated with an increased risk of stroke. Additionally, patients with AS did not have a higher risk of low on-treatment DBP or safety outcomes than patients without AS.

According to the GBD 2019 Stroke Collaborators [24], stroke remains the second-leading cause of death and the third-leading cause of death and disability combined worldwide. Recent evidence has shown that AS, which is common in patients with hypertension, is associated with adverse clinical outcomes including stroke [8], myocardial infarction [25], heart failure [26], and cardiovascular and all-cause death [7]. The results of our study showed a higher risk of stroke in patients with than without AS, which is consistent with previous studies.

With aging, large elastic arteries such as the aorta become stiffer, and this is accompanied by histological and biochemical changes of the arterial wall [27]. Vascular stiffness is linearly related to age in both normotensive and hypertensive subjects [28]. Given that AS is common in the elderly population [28], the balance of risks and benefits from intensive SBP treatment should be carefully evaluated according to the AS status of these patients. Previous analyses of the SPRINT trial have shown that intensive treatment significantly attenuates increases in the cfPWV and aortic elastance index, suggesting that attenuating AS progression might be one of the mechanisms underlying the cardiovascular benefit obtained from intensive SBP intervention [29, 30]. However, previous researchers did not investigate the influence of the baseline PWV status on the effects of intensive SBP treatment. Our study is the first to investigate the influence of baseline AS on the effect of intensive SBP treatment in a rigorously conducted randomized controlled trial in which the patients were under strict SBP control.

SBP rises continuously with increasing age, whereas DBP reaches a plateau at 50 to 60 years and decreases thereafter [31]. The DBP reduction occurs in parallel with increasing AS [32]. As previously reported, DBP of <70 mmHg is associated with an increased risk of coronary heart disease and heart failure [14–16, 33]. Patients with stiffening arteries might be more susceptible to developing low DBP and hypoperfusion to achieve the intensive SBP target. In this study, we found that the incidence of low on-treatment DBP (<70 mmHg) was higher in the intensive treatment group than in the standard treatment group. However, within the intensive treatment group, no difference in the risk of low on-treatment DBP was observed between the AS and non-AS groups, which indicates that AS does not aggravate the risk of low DBP during treatment when targeting lower SBP.

With respect to the cardiovascular benefits, we found that compared with the non-AS group, patients with AS at baseline showed a 1.70-fold higher risk for stroke among patients in this study. These results are consistent with a study conducted by Song et al. [34], which demonstrated a significant and independent association of AS with the risk of stroke even among individuals with controlled hypertension. These findings provide evidence of a residual risk of stroke attributed to AS in patients with good control of hypertension. Notably, our study showed no interaction between baseline AS status and SBP treatments on cardiovascular events including stroke and all-cause death, and the beneficial effects of intensive SBP treatment were similar in the AS and non-AS groups. There was also no suggestion of heterogeneity of the HRs for safety outcomes, including renal outcomes, among patients with AS or non-AS at baseline, indicating that the safety risk due to intensive SBP treatment was not aggravated in AS group. We suggested that, regardless of baseline AS status, intensive SBP treatment appeared beneficial to cardiovascular outcomes, and we did not observe higher risk brought by intensive SBP treatment in patients with AS at baseline.

A strength of the present study was our ability to examine the role of the baseline AS status on the treatment effect with the use of a randomized comparison. Other strengths include the ability to examine the association between the AS status and cardiovascular risks among patients with rigorously controlled hypertension as well as the large sample size with baPWV measurements. In addition, the well-organized and carefully recorded data of the STEP trial add to the reliability of our results. However, this study also has several limitations. First, more than 1000 participants lacked baPWV data, impairing the validity of a randomized comparison. Second, we did not use carotid-femoral aortic pulse wave velocity (cfPWV), which is considered the gold standard to assess the status of arterial stiffness. However, evidence has suggested that the predictive value of baPWV is similar to that of cfPWV for predicting clinical outcomes [19]. BaPWV also reflects structural and functional stiffness of the arterial wall, and baPWV simplifies the procedure and provides better reproducibility, making it more suitable for large cohort [35]. A strong association between baPWV and cfPWV (correlation coefficient, 0.73) was detected by a previous study [19], suggesting that baPWV could be used to measure arterial stiffness. Third, as a post hoc study of a randomized controlled trial, the statistical power could be insufficient. Because there is only about half of the whole population in each subgroup, the statistical power of the analysis was diminished, especially in AS group. Since this was an exploratory research, a new trial specifically designed was needed to further answer this question.

## Conclusions

In conclusion, among patients at 60 to 80 years of age with hypertension, the beneficial effects of an intensive SBP target of 110 to 130 mm Hg compared with a standard SBP target of 130 to 150 mm Hg on cardiovascular events were similar among those in the AS group and the non-AS group.

## Supplementary Information

**Additional file 1: Table S1.** Baseline characteristics for participants included in this study compared to the rest of the STEP cohort. **Table S2.** Effect of SBP intervention on cardiovascular outcomes, low on-treatment DBP, and safety outcomes. **Table S3.** Effect of baseline baPWV groups (AS versus non-AS) on cardiovascular outcomes via univariable and multivariable Cox proportional hazard regression. **Table S4.** Effect of per SD changes in baseline baPWV on cardiovascular outcomes via univariable and multivariable Cox proportional hazard regression. **Table S5.** Effect of baseline baPWV on safety outcomes via univariable and multivariable logistic regression. **Table S6.** Effects of SBP intervention on safety outcomes within AS and non-AS groups via multivariable logistic regression.

## Data Availability

The data that support the findings of this study will be publicly available 3 years after the completion of the STEP trial. Data are available from the corresponding author upon reasonable request and with permission of the corresponding author.

## Abbreviations

AS: Arterial stiffness
baPWV: Brachial-ankle pulse wave velocity
BP: Blood pressure
cfPWV: Carotid-femoral pulse wave velocity
CI: Confidence interval
CKD: Chronic kidney disease
DBP: Diastolic blood pressure
eGFR: Estimated glomerular filtration rate
HR: Hazard ratio
MACE: Major cardiovascular events
MAP: Mean arterial pressure
OR: Odds ratio
PP: Pulse pressure
SBP: Systolic blood pressure
