# Supplementary material for: Influence of baseline arterial stiffness on effects of intensive compared with standard blood pressure control: a post hoc analysis of the STEP trial
Source: BMC Med. 2022 Oct 20;20:358. doi: 10.1186/s12916-022-02556-1 (PMC9583479; doi:10.1186/s12916-022-02556-1)
Supplement: Supplementary file 1 — Additional file 1: Table S1. Baseline characteristics for participants included in this study compared to the rest of the STEP cohort. Table S2. Effect of SBP intervention on cardiovascular outcomes, low on-treatment DBP, and safety outcomes. Table S3. Effect of baseline baPWV groups (AS versus non-AS) on cardiovascular outcomes via univariable and multivariable Cox proportional hazard regression. Table S4. Effect of per SD changes in baseline baPWV on cardiovascular outcomes via univariable and multivariable Cox proportional hazard regression. Table S5. Effect of baseline baPWV on safety outcomes via univariable and multivariable logistic regression. Table S6. Effects of SBP intervention on safety outcomes within AS and non-AS groups via multivariable logistic regression. [file 12916_2022_2556_MOESM1_ESM.docx]

**Additional file 1**

**TableS1.** Baseline characteristics for participants included in this study compared to the rest of the STEP cohort

|  | **Included** | **Excluded** | **P value** |
| --- | --- | --- | --- |
| **N (%)** | 6865 (80.66) | 1646 (19.34) |  |
| **Age, years** | 66.14±4.75 | 66.65±5.1 | <0.001 |
| **Female, n (%)** | 3676 (53.55) | 876 (53.22) | 0.83 |
| **BMI, kg/m2** | 25.61±3.18 | 25.39±3.12 | 0.01 |
| **HR, bpm** | 73.55±10.42 | 73.44±9.94 | 0.70 |
| **BP, mmHg** |  |  |  |
| **SBP** | 146.51±16.81 | 144.16±15.73 | <0.001 |
| **DBP** | 82.59±10.56 | 82.01±10.62 | 0.05 |
| **PP** | 63.92±14.59 | 62.15±13.94 | <0.001 |
| **MAP** | 103.9±11.01 | 102.73±10.7 | <0.001 |
| **Physical activity, times/week, n (%)** |  |  | 0.89 |
| **≥ 3** | 4443 (64.72) | 1073 (65.19) |  |
| **1-2** | 1385 (20.17) | 332 (20.17) |  |
| **0** | 1037 (15.11) | 241 (14.64) |  |
| **Smoking status, n (%)** |  |  | 0.38 |
| **Current** | 1092 (15.95) | 280 (17.06) |  |
| **Former** | 812 (11.86) | 204 (12.43) |  |
| **Never** | 4944 (72.20) | 1157 (70.51) |  |
| **Drink status, n (%)** |  |  | 0.51 |
| **Current** | 1799 (26.27) | 443 (27.01) |  |
| **Former** | 355 (5.18) | 94 (5.73) |  |
| **Never** | 4694 (68.55) | 1103 (67.26) |  |
| **Fasting blood glucose, mmol/L** | 6.09±1.64 | 5.98±1.79 | 0.02 |
| **Total cholesterol, mmol/L** | 4.89±1.09 | 4.87±1.06 | 0.61 |
| **LDL cholesterol, mmol/L** | 2.69±0.87 | 2.65±0.88 | 0.11 |
| **HDL cholesterol, mmol/L** | 1.25±0.31 | 1.27±0.3 | 0.20 |
| **Triglyceride, mmol/L** | 1.59±1.1 | 1.63±1.01 | 0.29 |
| **Serum uric acid, mmol/L** | 347.95±89.39 | 348.07±90.21 | 0.97 |
| **eGFR, ml/min** | 109.11±23.85 | 109.75±25.31 | 0.36 |
| **eGFR<60 ml/min, n (%)** | 100 (1.70) | 25 (1.67) | 1.00 |
| **Medical history, n (%)** |  |  |  |
| **Diabetes mellitus** | 1345 (19.59) | 282 (17.13) | 0.02 |
| **CVD History** | 1219 (17.84) | 268 (16.56) | 0.15 |
| **Antihypertensive agents** |  |  |  |
| **No. of antihypertensive agents** | 1.48±0.67 | 1.24±0.73 | <0.001 |
| **Not using antihypertensive agents, n (%)** | 241 (3.51) | 231 (14.03) | <0.001 |
| **Use of ARB, n (%)** | 4242 (61.79) | 855 (51.94) | <0.001 |
| **Use of CCB, n (%)** | 5168 (75.28) | 1062 (64.52) | <0.001 |
| **Use of statins, n (%)** | 1347 (19.62) | 289 (17.56) | 0.06 |
| **Use of aspirin, n (%)** | 621 (9.05) | 109 (6.62) | 0.002 |
| **FRS ≥15%, n (%)** | 4569 (78.04) | 1118 (74.88) | 0.01 |
| Abbreviations: AS, arterial stiffness; BMI, body mass index; HR, heart rate; SBP, systolic blood pressure; DBP, diastolic blood pressure; PP, pulse pressure; MAP, mean arterial pressure; baPWV, brachial-ankle pulse wave velocity; FBG, fasting blood glucose; TC, total cholesterol; LDL-C, low-density lipoprotein cholesterol; HDL-C, high-density lipoprotein cholesterol; TG, triglyceride; UA, uric acid; eGFR, estimated glomerular filtration rate; CVD, cardiovascular disease; ARB, angiotensin receptor blocker; CCB, calcium channel blocker; FRS, Framingham risk score. | | | |

**TableS2.** Effect of SBP intervention on cardiovascular outcomes, low on-treatment DBP, and safety outcomes

|  | **Intensive Treatment** | **Standard Treatment** | **Hazard Ratio or Odds Ratio* (95% CI)** | **P value** |
| --- | --- | --- | --- | --- |
| **N** | 3444 | 3421 |  |  |
| **Primary outcome†, n (%)** | 103 (2.99) | 145 (4.24) | 0.69 (0.54-0.90) | 0.01 |
| **Secondary outcomes** |  |  |  |  |
| Stroke, n (%) | 31 (0.90) | 58 (1.70) | 0.54 (0.35-0.84) | 0.01 |
| MACE‡, n (%) | 73 (2.12) | 97 (2.84) | 0.72 (0.53-0.98) | 0.04 |
| ACS | 42 (1.22) | 55 (1.61) | 0.76 (0.50-1.14) | 0.19 |
| Death from any causes, n (%) | 43 (1.25) | 38 (1.11) | 1.15 (0.74-1.78) | 0.54 |
| **Safety outcomes** |  |  |  |  |
| Hypotension, n (%) | 134 (3.89) | 101 (2.95) | 1.37 (1.05-1.79) | 0.02 |
| Dizziness, n (%) | 42 (1.22) | 38 (1.11) | 1.09 (0.70-1.71) | 0.69 |
| Fracture, n (%) | 10 (0.29) | 16 (0.47) | 0.62 (0.27-1.36) | 0.24 |
| Syncope, n (%) | 4 (0.12) | 0 (0) | - | - |
| Renal outcome§, n (%) | 41 (1.19) | 54 (1.58) | 0.74 (0.48-1.11) | 0.14 |
| **Low on-treatment DBP, n/total (%)** | 214/3059 (7.00) | 77/3038 (2.53) | 3.34 (2.51-4.45) | <0.001 |
| Multivariable model was adjusted for clinical centers, age, and sex, baseline MAP level, baseline glucose level, baseline LDL cholesterol level, baseline antihypertensive agent type, physical activity frequency, smoking status, and drinking status. Missing values of physical activity frequency, smoking status, drinking status (n = 17, 0.26%), baseline glucose level (n=214, 3.12%), baseline LDL cholesterol level (n=178, 2.59%) were added via multiple imputation.  Abbreviations: SBP, systolic blood pressure; DBP, diastolic blood pressure; CI, confidence interval; MACE, major cardiovascular events.  *Hazard ratios were calculated for primary and secondary outcomes via Cox hazard regression models. Odds ratios were calculated for safety outcomes and the incidence of low on-treatment DBP via logistic regression.  †Primary outcome contains the first occurrence of stroke (ischemic or hemorrhagic), acute coronary syndrome (myocardial infarction and hospitalization for unstable angina), acute decompensated heart failure, coronary revascularization (percutaneous coronary intervention or coronary artery bypass grafting), atrial fibrillation, or death of cardiovascular causes.  ‡MACE was a composite of first occurrence of acute coronary syndrome, acute decompensated heart failure, coronary revascularization, and death of cardiovascular causes.  §Renal outcome was a composite of ≥50% decrease in estimated glomerular filtration rate (eGFR) in patients with chronic kidney disease (CKD) at baseline, ≥30% decrease in eGFR to <60 mL/min/1.73 m2 in patients without CKD at baseline, or serum creatinine increase of >1.5 mg/dL in men or >1.3 mg/dL in women. | | | | |

**TableS3.** Effect of baseline baPWV groups (AS versus non-AS) on cardiovascular outcomes via univariable and multivariable Cox proportional hazard regression

|  | **Non-AS** | **AS** | **Hazard Ratio (95% CI)** | **P value** |
| --- | --- | --- | --- | --- |
| **N** | 3818 | 3047 |  |  |
| **Primary outcome*, n (%)** | 117 (3.06) | 131 (4.30) |  |  |
| Crude model |  |  | 1.40 (1.09-1.79) | 0.01 |
| Model 1 |  |  | 1.24 (0.96-1.60) | 0.11 |
| Model 2 |  |  | 1.26 (0.97-1.63) | 0.08 |
| **Stroke, n (%)** | 35 (0.92) | 54 (1.77) |  |  |
| Crude model |  |  | 1.92 (1.26-2.95) | 0.003 |
| Model 1 |  |  | 1.77 (1.14-2.76) | 0.01 |
| Model 2 |  |  | 1.70 (1.09-2.65) | 0.02 |
| **MACE†, n (%)** | 87 (2.28) | 83 (2.72) |  |  |
| Crude model |  |  | 1.19 (0.88-1.61) | 0.26 |
| Model 1 |  |  | 1.04 (0.77-1.42) | 0.79 |
| Model 2 |  |  | 1.09 (0.80-1.49) | 0.57 |
| **ACS, n (%)** | 50 (1.31) | 47 (1.54) |  |  |
| Crude model |  |  | 1.17 (0.78-1.74) | 0.45 |
| Model 1 |  |  | 1.07 (0.71-1.61) | 0.74 |
| Model 2 |  |  | 1.12 (0.75-1.68) | 0.59 |
| **Death from any causes, n (%)** | 35 (0.92) | 46 (1.51) |  |  |
| Crude model |  |  | 1.64 (1.06-2.55) | 0.03 |
| Model 1 |  |  | 1.26 (0.80-1.98) | 0.32 |
| Model 2 |  |  | 1.21 (0.70-1.92) | 0.41 |
| Model 1 was adjusted for age and sex. Model 2 was adjusted for intervention groups, clinical centers, age, and sex, baseline MAP level, baseline glucose level, baseline LDL cholesterol level, baseline antihypertensive agent type, physical activity frequency, smoking status, and drinking status. Missing values of physical activity frequency, smoking status, drinking status (n = 17, 0.26%), baseline glucose level (n=214, 3.12%), baseline LDL cholesterol level (n=178, 2.59%) were added via multiple imputation.  Abbreviations: baPWV, brachial-ankle pulse wave velocity; MACE, major cardiovascular events; AS, arterial stiffness; CI, confidence interval; ACS, acute coronary syndrome; MACE, major adverse cardiac events.  *Primary outcome contains the first occurrence of stroke (ischemic or hemorrhagic), acute coronary syndrome (myocardial infarction and hospitalization for unstable angina), acute decompensated heart failure, coronary revascularization (percutaneous coronary intervention or coronary artery bypass grafting), atrial fibrillation, or death of cardiovascular causes.  †MACE was a composite of first occurrence of acute coronary syndrome, acute decompensated heart failure, coronary revascularization, and death of cardiovascular causes. | | | | |

**TableS4.** Effect of per SD changes in baseline baPWV on cardiovascular outcomes via univariable and multivariable Cox proportional hazard regression

|  | **Hazard Ratio (95% CI) per SD change in baseline baPWV** | **P value** |
| --- | --- | --- |
| **Primary outcome*** |  |  |
| Crude model | 1.14 (1.02-1.27) | 0.02 |
| Model 1 | 1.06 (0.95-1.19) | 0.33 |
| Model 2 | 1.07 (0.95-1.20) | 0.26 |
| **Stroke** |  |  |
| Crude model | 1.31 (1.09-1.58) | 0.004 |
| Model 1 | 1.25 (1.03-1.52) | 0.02 |
| Model 2 | 1.22 (1.00-1.49) | 0.05 |
| **MACE†** |  |  |
| Crude model | 1.05 (0.92-1.20) | 0.48 |
| Model 1 | 0.97 (0.85-1.11) | 0.66 |
| Model 2 | 0.99 (0.86-1.13) | 0.99 |
| **ACS** |  |  |
| Crude model | 1.01 (0.85-1.20) | 0.95 |
| Model 1 | 0.95 (0.80-1.14) | 0.61 |
| Model 2 | 0.97 (0.82-1.16) | 0.78 |
| **Death from any causes** |  |  |
| Crude model | 1.35 (1.11-1.65) | 0.003 |
| Model 1 | 1.18 (0.95-1.45) | 0.13 |
| Model 2 | 1.16 (0.94-1.44) | 0.17 |
| Model 1 was adjusted for age and sex. Model 2 was adjusted for intervention groups, clinical centers, age, and sex, baseline MAP level, baseline glucose level, baseline LDL cholesterol level, baseline antihypertensive agent type, physical activity frequency, smoking status, and drinking status. Missing values of physical activity frequency, smoking status, drinking status (n = 17, 0.26%), baseline glucose level (n=214, 3.12%), baseline LDL cholesterol level (n=178, 2.59%) were added via multiple imputation.  Abbreviations: baPWV, brachial-ankle pulse wave velocity; MACE, major cardiovascular events; AS, arterial stiffness; CI, confidence interval; SD, standard deviation; ACS, acute coronary syndrome; MACE, major adverse cardiac events.  *Primary outcome contains the first occurrence of stroke (ischemic or hemorrhagic), acute coronary syndrome (myocardial infarction and hospitalization for unstable angina), acute decompensated heart failure, coronary revascularization (percutaneous coronary intervention or coronary artery bypass grafting), atrial fibrillation, or death of cardiovascular causes.  †MACE was a composite of first occurrence of acute coronary syndrome, acute decompensated heart failure, coronary revascularization, and death of cardiovascular causes. | | |

**TableS5.** Effect of baseline baPWV on safety outcomes via univariable and multivariable logistic regression

|  | **Non-AS** | **AS** | **Odds Ratio (95% CI)** | **P value** |
| --- | --- | --- | --- | --- |
| **N** | 3818 | 3047 |  |  |
| **Hypotension, n (%)** | 180 (4.71) | 55 (1.81) | 0.37 (0.27-0.50) | <0.001 |
| **Dizziness, n (%)** | 53 (1.39) | 27 (0.89) | 0.71 (0.43-1.15) | 0.18 |
| **Fracture, n (%)** | 7 (0.18) | 19 (0.62) | 3.01 (1.27-7.94) | 0.02 |
| **Syncope, n (%)** | 3 (0.08) | 1 (0.03) | 0.58 (0.03-5.24) | 0.65 |
| **Renal outcome*****, n (%)** | 42 (1.10) | 53 (1.74) | 1.40 (0.92-2.16) | 0.12 |
| Multivariable model was adjusted for intervention groups, clinical centers, age, and sex, baseline MAP level, baseline glucose level, baseline LDL cholesterol level, baseline antihypertensive agent type, physical activity frequency, smoking status, and drinking status. Missing values of physical activity frequency, smoking status, drinking status (n = 17, 0.26%), baseline glucose level (n=214, 3.12%), baseline LDL cholesterol level (n=178, 2.59%) were added via multiple imputation.  Abbreviations: baPWV, brachial-ankle pulse wave velocity; AS, arterial stiffness; CI, confidence interval.  *Renal outcome was a composite of ≥50% decrease in estimated glomerular filtration rate (eGFR) in patients with chronic kidney disease (CKD) at baseline, ≥30% decrease in eGFR to <60 mL/min/1.73 m^2^ in patients without CKD at baseline, or serum creatinine increase of >1.5 mg/dL in men or >1.3 mg/dL in women. | | | | |

**TableS6.** Effects of SBP intervention on safety outcomes within AS and non-AS groups via multivariable logistic regression

|  | **Non-AS** | | | | **AS** | | | | **P for interaction*** |
| --- | --- | --- | --- | --- | --- | --- | --- | --- | --- |
|  | **Intensive Treatment** | **Standard Treatment** | **Odds Ratio (95% CI)** | **P value** | **Intensive Treatment** | **Standard Treatment** | **Odds Ratio (95% CI)** | **P value** |  |
| **N** | 1980 | 1838 |  |  | 1464 | 1583 |  |  |  |
| **Hypotension, n (%)** | 100 (5.05) | 80 (4.35) | 1.20 (0.89-1.63) | 0.24 | 34 (2.32) | 21 (1.33) | 1.85 (1.07-3.26) | 0.03 | 0.21 |
| **Dizziness, n (%)** | 28 (1.41) | 25 (1.36) | 1.06 (0.61-1.84) | 0.85 | 14 (0.96) | 13 (0.82) | 1.15 (0.53-2.50) | 0.72 | 0.80 |
| **Fracture, n (%)** | 2 (0.10) | 5 (0.27) | 0.35 (0.05-1.65) | 0.21 | 8 (0.55) | 11 (0.69) | 0.80 (0.30-1.98) | 0.63 | 0.42 |
| **Syncope, n (%)** | 3 (0.15) | 0 (0) | - | - | 1 (0.07) | 0 (0) | - | - | - |
| **Renal outcome†, n (%)** | 19 (0.96) | 23 (1.25) | 0.73 (0.39-1.36) | 0.32 | 22 (1.50) | 31 (1.96) | 0.77 (0.43-1.33) | 0.35 | 0.90 |
| Multivariable model was adjusted for clinical centers, age, and sex, baseline MAP level, baseline glucose level, baseline LDL cholesterol level, baseline antihypertensive agent type, physical activity frequency, smoking status, and drinking status. Missing values of physical activity frequency, smoking status, drinking status (n = 17, 0.26%), baseline glucose level (n=214, 3.12%), baseline LDL cholesterol level (n=178, 2.59%) were added via multiple imputation.  Abbreviations: SBP, systolic blood pressure; AS, arterial stiffness; CI, confidence interval.  * P for interaction was calculated by the multiplicative interaction between the baseline AS and non-AS groups and SBP intervention for the incidence of each component of safety outcomes.  **†**Renal outcome was a composite of ≥50% decrease in estimated glomerular filtration rate (eGFR) in patients with chronic kidney disease (CKD) at baseline, ≥30% decrease in eGFR to <60 mL/min/1.73 m^2^ in patients without CKD at baseline, or serum creatinine increase of >1.5 mg/dL in men or >1.3 mg/dL in women. | | | | | | | | | |
